# Diseases of the Digestive Organs in Infancy and Childhood

**Published:** 1886-07

**Authors:** 


### Bibliographical.

DISEASES OF THE DIGESTIVE ORGANS IN INFANCY AND CHILDHOOD, with chapters on the investigations of disease and on the general management of children. By Louis Starr, M. D., Clinical Professor of Diseases of Children in the University of Pennsylvania, etc.. with colored plates and other illustrations.

This excellent treatise, the most recent publication on diseases of children and their management, is a valuable contribution to medical literature, and cannot fail to be a valuable guide and assistant to both the practicing physician and the student. Commencing with the investigation of disease it comprehensively treats affections of the mouth and throat, of the stomach and intestines, of the mesenteric glands, of the liver, of the peritoneum concluding with an excellent treatise on the general management of children, which must be of great service to all interested.

The publishers, Messrs. P. Blakiston, Son & Co., Philadelphia, have presented the present edition of this work in handsome style and large type with finely executed engravings.

